# Prescribing pattern of psychotropic medications in child psychiatric practice in a mental referral hospital in Botswana

**DOI:** 10.11604/pamj.2017.26.83.11212

**Published:** 2017-02-21

**Authors:** Anthony Olashore, James Ayugi, Philip Opondo

**Affiliations:** 1 Department of Psychiatry, University of Botswana Medical School, Gaborone, Botswana

**Keywords:** Prescribing pattern, psychotropic medications, child psychiatry, mental referral, Botswana, review

## Abstract

**Introduction:**

There is a growing preference for psycho-pharmacological therapy over non-pharmacological care. The prescription pattern and the choice of psychotropic medications vary in different settings. Whilst newer agents and rational prescribing are favored in the more specialized settings, the pattern remains unclear in less specialized units, largely due to lack of data. The aims were to conduct a treatment audit in the only mental referral hospital in Botswana, which is a non-specialized child and adolescent care setting and see how it conforms to best practice.

**Methods:**

A retrospective audit which involved the extraction of socio-demographic and clinical information from the records of patients who were ≤ 17 years and seen from January 1, 2012-July 31, 2016.

**Results:**

A total of 238 files were used for this report. Mean age (SD) was 12.41 (4.1) years. Of the 120 (50.4%) patients who had pharmacological intervention, only 85(70.8%) had monotherapy. The most commonly prescribed psychotropic agents were antipsychotics (40%). Off-label use of antipsychotics and polypharmacy were 31.2% and 29.2% respectively.

**Conclusion:**

The level of conformity to standard practice in terms of psychotropic prescribing in our setting is consistent with the reports from developed countries where more specialized care ostensibly exists. Further studies will be necessary to determine the scope of psychotropic use.

## Introduction

Psychotropic medications use other than stimulants have not been adequately studied in children in terms of benefit and safety. Their use in children is mostly based on extrapolation of information from adult studies [1]. There is a growing preference for the use of psychotropic medications over other non-pharmacological interventionsin child and adolescent mental care [1–3]. Factors majorly responsible for this preference include severity or type of psychiatric disorder, the need for quick symptom relief and lack of multidisciplinary care, particularly in the low resourced countries [1, 2]. Patterns of prescribing and the choice of psychotropic medications vary in different settings [4]. Whilst newer agents and rational prescribing (justifiable use) are favored in specialized settings [1–3, 5], the pattern remains largely unclear in settings without specialized care units for children, mainly due to lack of data [6]. In Europe and America for instance, where specialized child and adolescent health care services are available, psychosocial management is more preferred, especially for mild to moderate conditions [1–3, 5]. Newer agents such as Risperidone, olanzapine, fluoxetine are preferred to older ones like haloperidol and amitriptyline [1, 2, 7]. Nonetheless, they have also reported increase in rate of off-label use of psychotropic medications (psychotropic prescription outside its approved indications) [2, 7, 8] and polypharmacy among children and adolescent in these centers [2, 9–11]. Studies conducted in Africa among adult population, though scanty, have likewise shown a high rate of off-label use of psychotropics low preference for newer agents due to cost and polypharmacy [12, 13]. It is however not clear the extent to which these apply to children and adolescents in centers without specialized care. Botswana spends about 5.8% of its Gross Domestic Product (GDP) on health and provides free medical for all the citizens, but only one percent of its total health budget goes to mental health care [14]. It operates three tiers of health care system (primary, secondary and tertiary), with mental health being part of all levels but has only one mental referral (tertiary) hospital which majorly provides services for adult and some children with mental conditions. Child and adolescent services are thus being provided by general adult mental specialists who are currently less than 10 in a population of 2.1 million people [15] and no evaluation of the effectiveness of psycho-pharmacological therapies currently on offer has been done. We therefore decided to conduct a treatment audit in the mental tertiary hospital in Botswana, which is a non-specialized child and adolescent care setting, and see how it conforms to rational drug prescription pattern in the developed countries. It was also hoped that the findings of this study would lay a foundation for biomedical research in pediatric mental health prescribing in this setting.

## Methods

We conducted a retrospective audit of 238 children and adolescents who attended Sbrana psychiatric hospital (SPH) from the first of January, 2012 when the hospital record could easily be accessed to the thirty-first of July, 2016 when we decided to conduct this audit.

**Location of study:** SPH is located in Lobatse, South-Western district of Botswana and is staffed by six (6) general adult psychiatrists, ten (10) medical officers and over one hundred (100) nurses. SPH is a 300-bed facility which receives referrals (both formal and informal) from all the health care institutions in Botswana. The hospital policy ensures a proper documentation and uses a computerized record keeping system, which make data extraction for research purposes convenient.

**Sampling and data collection procedure:** The study was approved by the University of Botswana ethical committee and permission was obtained from the SPH management. A semi-structured questionnaire was employed for the extraction of information such as Socio-demographic data (age and gender), diagnosis (using ICD -10 criteria) and management from the files of all patients under 18 years. This age cut off point was selected based on definition of a child by the convention on the right of the child (CRC). This involved all the in-patients and out-patients managed from the 1^st^ of January 2012 till the 31^st^ of July, 2016. Every psychotropic medication given throughout the management regardless of their mode of care (whether on admission or during outpatient visit) were recorded. A patient is considered to receive multi-drug treatment when more than two medications were given at the same time. This pro-forma was designed by the researchers based on previous studies [2, 16]. Data extraction was done by two psychiatrists from the hospital who agreed on every information needed before they were recorded in the pro-forma. However, files which information could not be agreed on or with incomplete documentation on variables of interest were excluded from the analysis to minimize coding bias.

**Data analysis:** Statistical analysis was done using Statistical Package for Social Sciences version 16 (SPSS -16). Frequency tables were used for descriptive statistics such as socio-demographics and clinical variables.

## Results

A total of 238 case files were used for this report. Mean age (sd) was 12.41 (4.1). Most of the patients were above 10 years of age (66.4%), and were males (60.5%). The disorder commonly diagnosed were hyperkinetic disorder (25.2%), conduct disorder [CD] (18.5%), psychosis (14.7%), epilepsy (11.8%) and bipolar (2.5%), either as a single or multiple diagnosis. Forty-seven (19.7%) of these patients have had an inpatient care at least once in the course of their treatments, while majority (65%) had been reviewed at least once by a general psychiatrist. Almost half (49.6%) had only non-pharmacological treatments which included psychological treatment, occupational therapy, family intervention and so on (Table 1). Only 120 (50.4%) patients had pharmacological intervention with or without psychotherapy; 85 (70.8%) had monotherapy, 30 (25%) had 2 medications, while 4(3.3%) and 1(0.8%) had 3 and 4 medications (at the same time) respectively (Table 2). Of the 120 patients who had pharmacotherapy, 45(37.5%) had stimulants whether as a monotherapy or poly-therapy, 48 (40%) had antipsychotics. When the different types of antipsychotics (i.e., olanzapine, risperidone, haloperidol etc.) used throughout the treatment period (whether for few days or years) were considered, it was observed that antipsychotic was used 61(50.8%) times (Table 3); risperidone accounted for 49.1%, while olanzapine 14.8%. Haloperidol was used in 26.2% of cases, while only three (4.9%) had depot (injectable long acting) form of conventional antipsychotic. Seventeen (14.2%) of those who had psychotropic medications had antidepressants: tricyclic antidepressant was given to 12(70.6%) patients, selective serotonin re-uptake inhibitor was used in by the remaining 5(29.4%) (Table 3). Anticonvulsants (25%) which include Sodium valproate and carbamazepine were prescribed majorly for epilepsy and a few cases of bipolar. Benzodiazepines was administered to 14(11.6%) patients for sleep related problems, while *promethazine as pro re nata* was administered to 3(2.5%) patients during in-patient care (Table 2). The rate of ‘off-label’ prescribing among 48 who had antipsychotic medication (measured by use for unlicensed purposes) was 54.2%.

**Table 1:** Clinical variables of the patients

Clinical variables	N+	%
**Common diagnosis+**	**238**	**100**
ADHD	60	25.2
DBD	44	18.5
Psychosis	35	14.7
Epilepsy	28	11.8
Depressive disorder	18	7.6
Mode of care	238	100
In-patient	47	19.7
Out-patient	191	80.3
**Type of intervention**	**238**	**100**
Only pharmacological	45	18.9
Non-pharmacological only	118	49.6
Both	75	31.5
**Prescribing pattern^a^**	**120**	**100**
Monotherapy	85	70.8
Poly-therapy	35	29.2
**Specialist care++**	**238**	**100**
Given	156	65.5
Not given	82	34.5

a N = not equal to 238 due to missing data, N = 238.

+ Only common multiple diagnosis were reported, so N is not equal to 238.

++ Specialist care refers to general adult psychiatrists’ care

**Table 2:** Frequency of those who had psychotropic medications

Monotherapy	120	100
Antipsychotic	21	17.5
Antidepressant	10	8.3
Stimulants	26	21.8
Anticonvulsant	15	12.5
Sedatives	13	10.8
**Combination therapy**		
Antipsychotic + antidepressant	6	5.0
Antipsychotic + stimulants	7	5.8
Antipsychotic + anticonvulsant	4	3.3
Antipsychotic + anticholinergic	2	1.7
Antipsychotic + promethazine (*pro re nata*)	3	2.5
Anticonvulsant + stimulant	7	5.8
Antidepressant + anticonvulsant	1	0.8
Antipsychotic + anticholinergics + stimulant	1	0.8
Antipsychotic +anticonvulsant + stimulant	3	2.5
Antipsychotics + stimulants + sedative + anticonvulsant	1	0.8

^+^N is not equal to 238 and represent only those who had pharmacotherapy +/- psychotherapy

**Table 3:** Types of psychotropic medications

Drug type	N	%
Antipsychotics	61	100
Risperidone	30	49.1
Olanzapine	9	14.8
Quetiapine	3	4.9
Haloperidol	16	26.2
Flupenthixol	3	4.9
Antidepressants	17	100
Amitriptyline	12	70.6
Fluoxetine	5	29.4
Benzodiazepines	14	100
Diazepam	5	35.7
Lorazepam	9	64.3
Anticonvulsants	29	100
Sodium valproate	20	69.0
Carbamazepine	9	31.0

## Discussion

The current study presented a collection of data about the prescribing pattern among children and adolescents from a national mental hospital in Botswana. Being the only mental referral hospital in the country, the study provided an invaluable overview of psychotropic use among children with mental disorders in Botswana. The rate of non-pharmacological (49.5%) interventions such as counselling, family work, educational and occupational therapy intervention, is consistent with relevant literature [2, 3, 5, 16]. Antipsychotics were the most prescribed psychotropics (40%), either as a monotherapy or combined. Unlike a study conducted among adult patients in Nigeria [12, 13], where the use of the newer agents are less favored due to cost, atypical agents were more prescribed, with risperidone, olanzapine and quetiapine accounting for 68.8% of all antipsychotics. The current practice of child and adolescent mental care in our setting despite the shortage of specialists still follows the trend of atypical antipsychotic preference over the conventional ones as in the developed countries [2, 5, 16]. This is made possible by the fact that government is responsible for most of the healthcare funding, with free health care at all levels in Botswana and only about 5% being out of pocket [14]. Although atypical medications have not been adequately studied among children [2, 3], they have been recommended for use by the National Institute for Health and Care Excellence (NICE) guideline as first line in the treatment of psychosis in the pediatric population [17]. When used cautiously, they are associated with less risk of extrapyramidal side effects (EPSE), albeit their use should be balanced against the risk of significant metabolic effects [18]. Of the two commonly prescribed atypical antipsychotics in our setting; olanzapine and risperidone, the latter is preferred as the first line medication for psychosis or any other disorder for which antipsychotics are prescribed. This agrees with reports that risperidone is the most prescribed of all the second generation antipsychotics in children with psychotic conditions [2, 19, 20]. Moreover, metabolic side effects such as weigh gain, dyslipidemia to which children may be more vulnerable than their adult counterparts, are more associated with olanzapine [21]. Other reasons for this preference deserves further investigations. First generation antipsychotics such as haloperidol only accounted for 26.2% of antipsychotic medications used as in the studies from Europe and America [2, 22, 23], which explains the lower rate of EPSE observed and anticholinergic use (2.5%), compared to a report from another setting in Africa [6].

Methylphenidate (a stimulant), was another psychotropic used in 37.5% of cases. This psychotropic medication was originally licensed for use in pediatrics with hyperkinetic disorder and now being used in adult [1]. Its use in our setting was not only in line with the NICE guideline but in accord with other studies, where it was believed to be superior to psychological treatment alone [1, 2, 18]. The use of other medications like antidepressant and anticonvulsant among our sample were also in line with approved guidelines for various disorders such as depression and anxiety disorders, bipolar and epilepsy respectively [18]. Antipsychotic prescription outside its approved indications (off-label) was remarkable among others, accounting for 54.2% of its use and is comparable to a report from more specialized child care setting [2], thus suggesting that the practice is not restricted to settings without specialized care. The practice of using medications outside their licensed indications was previously known in adult; it is currently becoming a common practice in pediatric population [7, 24]. Disorders such CD and hyperkinetic disorder, which are externalizing in nature, are often misdiagnosed as psychotic disorders thus informing the use of antipsychotics [2]. For example in the current study, 13 (29.5%) of those who had hyperkinetic disorder were given antipsychotics in the course of management while half of the 44 patients with CD equally had antipsychotics. Nevertheless, only one 1 patient had co-occurring psychosis and CD, while none had hyperkinetic disorder and psychosis. It is therefore possible that these disorders were previously misdiagnosed, hence the reason for being given antipsychotic trial. Depot antipsychotic (flupenthixol) was used in three (4.9%) cases as in a previous study [2], but was restricted to older teens who had compliance issues, though it has not been licensed for use below the age of 18 years [2]. Benzodiazepines (which was used for sleep problems) prescribing was exclusively off-label, accounting for 11.6% of psychotropic use in the current study similar to a report from the United Kingdom [2]. Psychotropic medications e.g., antipsychotics and benzodiazepines have been used for various purposes for which they are not approved in psychiatry with good results. These include agitation, aggression, obsessive compulsive disorder, posttraumatic stress disorder and sleep problems [2, 7, 8]. There is therefore a need for more research into other potential benefits of psychotropic medications especially as it affect pediatric mental health.

Polypharmacy, defined as the prescription of two or more medications concurrently to a patient [9], is another notable feature in our study, as 29.2% rate was found. This is not peculiar to our center as authors from the developed [2, 9, 10] and developing countries [13, 25] have reported an increasing trend of multidrug treatment among children with mental conditions. In this audit, the most common pharmacological pair were antipsychotics plus stimulants and anticonvulsants plus stimulants, while the most common three combinations were antipsychotic-anticonvulsant-stimulant. Albeit the rationale behind the clinical decisions concerning psychotropic co-prescribing was not routinely documented, these combinations suggest that disorders which often presented with aggressiveness and externalizing behavioral symptoms are mostly responsible for polypharmacy in our setting as reported by an earlier author [10]. Polypharmacy is sometimes associated with risks which include adverse effects of drug interactions and increased toxicity, especially when liver enzyme inhibitors are part of the combinations [9]. This practice may not necessarily be an improper prescribing pattern as it is useful in some clinical situations, such as, the treatment of adverse effect of another agent, co-existing condition e.g., seizure and psychosis, immediate relief of symptoms while waiting for the main medication to act and so on. A consensus statement issued by some Psychiatrists in the United Kingdom rationalizes some cases of transitory polypharmacy, comprising making a gradual change from one psychotropic to another (Royal College of Psychiatrists, 1993) [9]. Moreover, one author is of the opinion that the “demerits of polypharmacy are not contained in ‘where’ the drugs are being used but in ‘how’ these drugs are being used” [9], further buttressing the fact that rational (justifiable use) polypharmacy could be beneficial. A new generation of research is required to guide optimal medication management and to identify the clinical situations where psychotropic co-prescribing is superior to monotherapy in terms of clinical outcomes.

**Recommendations:** in light of the above findings, we recommend more research on psychotropic medications use in children as this may reveal some of their latent benefits, thus widening the scope of their use in this age group. Prescription protocol, frequent training programs, and collaboration with the hospital pharmacy should be encouraged as this will improve adherence to rational prescribing, especially in the non-specialized care settings. In addition, more studies (multicenter) with larger sample will be required, to truly establish if our finding is a true reflection of the practice in Botswana and other non-specialized centers in Sub-Saharan Africa. **Limitations:** we were unable to ascertain the durations and the dosages of all the medications because of the documentation style in our setting, as these information would have been of great assistance when assessing conformity to standard practice. The generalizability of this study to other non-specialized centers is limited by the sample size.

## Conclusion

This study reveals an overview of prescribing pattern in a non-specialized child and adolescent care setting. Newer psychotropic agents with less adverse effect are more favored in this setting compared to the older agents. The practice of polypharmacy and off-label use of psychotropic is similar to what had been reported from settings with more specialized care. Thus, this data suggest some level of conformity to standard practice (concerning psychotropic prescribing) and a need for more biomedical research on the latent benefits of psychotropic medications in order to widen the scope of their use.

### What is known about this topic

- Prescribing pattern of psychotropics in specialized child and adolescent settings has been reported especially in the developed countries such as United States and United Kingdom;
- Prescribing pattern of psychotropics in general adult psychiatric practice has also been reported both in the developed and the developing countries.

### What this study adds

- Describes the pattern of prescribing psychotropic medications in Botswana which is an example of a non-specialized child mental health center and compare to those of the developed countries for the first time.
